# Growth patterns in patients with congenital adrenal hyperplasia analyzed by the QEPS growth model

**DOI:** 10.3389/fendo.2026.1736405

**Published:** 2026-06-05

**Authors:** Ruta Navardauskaite, Aimon Niklasson, Andreas F. M. Nierop, Aldina Pivodic, Rasa Verkauskiene, Anton Holmgren

**Affiliations:** 1 Department of Endocrinology, Lithuanian University of Health Sciences, Medical Academy, Kaunas, Lithuania; 2 Gothenburg Pediatric-Growth Research Centre (GP-GRC), Department of Pediatrics, Institute of Clinical Sciences, Sahlgrenska Academy, University of Gothenburg, Gothenburg, Sweden; 3 Muvara bv, Multivariate Analysis of Research Data, Leiderdorp, Netherlands; 4 APNC, Gothenburg, Sweden; 5 Department of Ophthalmology, Institute of Neuroscience and Physiology, The Sahlgrenska Academy, University of Gothenburg, Gothenburg, Sweden; 6 Institute of Endocrinology, Medical Academy, Lithuanian University of Health Sciences., Kaunas, Lithuania; 7 Department of Pediatrics, Halland Hospital, Halmstad, Sweden; 8 Department of Research and Development, Region Halland, Halmstad, Sweden

**Keywords:** adult height, childhood growth, congenital adrenal hyperplasia, growth patterns, pubertal growth, QEPS growth model

## Abstract

**Context:**

Patients with congenital adrenal hyperplasia (CAH) often face challenges in achieving their target adult height. The QEPS growth model, previously used for assessing healthy and pathological growth patterns, has not been applied to individuals with CAH.

**Objective:**

To evaluate growth patterns in patients with CAH using the QEPS growth model and to compare their growth characteristics with a healthy reference population.

**Design:**

A retrospective longitudinal study analyzing growth data collected from 1986 to 2008.

**Setting:**

The study was conducted in a single tertiary care center.

**Patients:**

The study included 25 patients (13 girls) with CAH, subtyped into salt wasting (SW; 12 boys, 8 girls) and simple virilizing (SV; 5 girls). Growth data were compared with a healthy reference cohort.

**Interventions:**

All patients were treated with hydrocortisone, and patients with CAH-SW received mineralocorticoids.

**Main outcome measures:**

Growth patterns were analyzed using the QEPS model, which includes specific early life growth (E-function), basic childhood growth (Q-function), and specific pubertal growth (P-function). Final adult height was compared with the reference population and parental heights.

**Results:**

CAH-SW boys and girls were longer at birth, exhibited reduced early-life growth, and had reduced puberty-specific growth (lower Pmax), resulting in shorter adult height (1.7 SDS). CAH-SV girls had earlier pubertal growth onset, also leading to reduced adult height (−1.6 SDS). Both groups showed similar basic childhood growth.

**Conclusions:**

Patients with CAH-SW displayed distinct growth patterns, including longer birth length but reduced specific early and pubertal growth, resulting in shorter adult height compared with reference populations and parental heights.

## 1 Introduction

Congenital adrenal hyperplasia (CAH) is a group of autosomal recessive disorders which represent one of the most common forms of inherited endocrine disorder, with an incidence of approximately 1 in 15,000 live births (1). Clinically, the classical form of CAH is divided to the generally more severe salt wasting (SW) form that may lead to life-threatening adrenal failure and the simple virilizing (SV) form. In both types of classical CAH, an enzyme deficiency in the adrenal glands leads to impaired cortisol biosynthesis (with or without aldosterone deficiency) and to excess androgen production. Patients with CAH receive life-long glucocorticoid (GC) treatment to correct the cortisol deficiency and prevent androgen overproduction; however, this treatment carries a high risk of side effects, one of which is short stature (2). Androgens are well known to be important for bone formation and growth during adrenarche (3). Individuals with CAH typically do not reach their adult height potential, which has largely been attributed to a combination of both overtreatment and undertreatment with GCs (4, 5). Excessive GC treatment directly inhibits growth during all growth phases, being infancy, childhood, and during puberty (6). Conversely, insufficient GC treatment leads to excessive adrenal androgen production which accelerates growth in childhood and early adolescence and increases the risk of earlier epiphyseal fusion and shorter adult height (1, 2). Not only androgens by promoting early epiphyseal fusion and consequent growth stunting in CAH but also aromatized estrogen products of adrenal androgens also contribute to growth alterations (1, 7).

Under normal conditions, pubertal growth follows an S-shaped course. Pubertal growth begins at a slow rate before accelerating to reach its highest rate in the middle of puberty at peak height velocity (PHV) after which the growth rate gradually decreases until the point at which epiphyseal fusion terminates longitudinal bone growth and growth stops. PHV generally occurs 1.5–2 years later in boys than in girls (8). The pubertal growth spurt is sex hormone driven, and closure of the epiphysis is triggered in both sexes by estrogens; in boys, through the conversion of testosterone to estrogen (9). Previous research has shown inconsistent results regarding the pubertal growth spurt in individuals with CAH. There is evidence from some studies that pubertal growth is impaired in children with CAH, whereas other studies found no difference in the magnitude of pubertal growth in individuals with CAH compared with a healthy population (10, 11). Some studies also suggest that the pubertal growth spurt starts earlier in children with CAH (11). Although Quadratic–Exponential–Pubertal–Stop (QEPS) modeling has been applied to several pediatric growth disorders, it has not previously been used to characterize growth trajectories in patients with classical CAH. Consequently, detailed phase-specific information on early growth, pubertal timing, and pubertal height gain in CAH remains limited.

**Figure 1:**
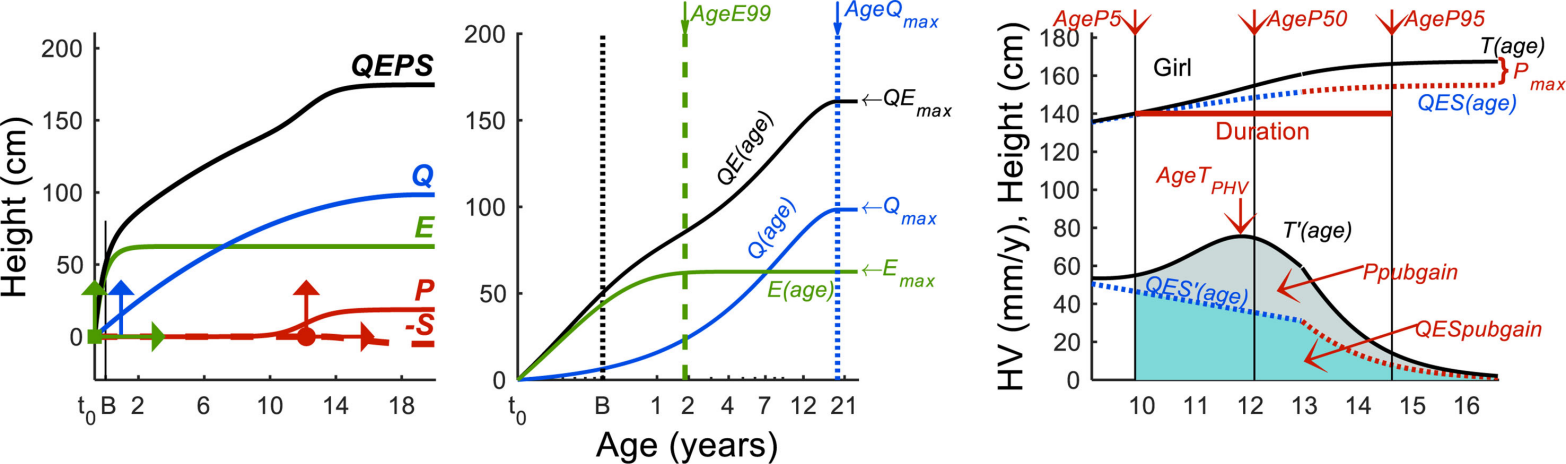
QEPS-growth model, total, prepubertal, and pubertal. Left: The four mathematical functions of the QEPS model that are combined to describe total gain in height (*QEPS*) from fetal life to adulthood; Quadratic (*Q*), Exponential (*E*), Pubertal (*P*), and Stop (*S*). B = birth, marked with a vertical line. t0 = approximately 6 weeks after conception. Middle: The vertical arrows at the top indicate from left to right AgeE99, the age in years where 99% of the E-function is achieved and *AgeQ_max_* the age in years when *Q_max_* is achieved. The three horizontal arrows at the right-hand side indicate the height-scale parameters, *E_max_* and *Q_max_* and their sum *QE_max_*. Right: Total height and height velocity estimated by the QEPS model. Onset, *AgeP5* as age at which 5% of the P-function growth is reached; mid puberty, *AgeP50* as age at which 50% of the P-function growth is reached; and end of pubertal growth, *AgeP95* as age at which 95% of the P-function growth is reached are marked with vertical lines. The duration of puberty is shown by the bold horizontal line. *AgeT_PHV_* indicates the age at peak height velocity of the total height function *T* from the QEPS model. The pubertal height gain is shown from AgeP5 to AgeP100 with marked areas in the height velocity graph; *Ppubgain* indicating the specific pubertal part and *QESpubgain* indicating the gain in height due to the pubertal growth of the *QES-*function. *Ppubgain* and *QESpubgain* add up to *Tpubgain*, the total pubertal gain in height.

The QEPS growth model analyzes longitudinal linear growth (length/height) trajectories by decomposing total growth into early life (E), childhood (Q), pubertal (P), and growth cessation (S) components. The model yields biologically interpretable parameters, including Emax, Qmax, Pmax, pubertal timing (AgeP5/50/95), and total pubertal height gain. The validated (12) QEPS model which is available today makes it possible to describe and analyze growth patterns in a more detailed way than previously (**Figure 1**) (13, 14). The QEPS model describes growth from fetal life to adult height using four mathematical functions; both the *E*- and *Q-functions* begin during fetal life and with the specific *E-function* mainly describing growth during early life—including infancy and the *Q-function* mainly describing childhood growth. Growth attributed to *Q-function* continues during the pubertal years and can be separated from the specific pubertal growth described by the *P-function*. The *S-function* ends the ongoing *Q-function*. The model is versatile, providing measures both in cm/years and in standard deviation scores (SDS) with individual confidence intervals (CI). Thereby, it allows detailed and comprehensive analysis of growth during early-life infancy, childhood, and adolescence in populations and children with disordered growth in comparison with healthy children (13, 14). Growth outcomes are expressed as SDS, representing deviation from the population mean relative to the reference standard deviation.

The aim of this study was to characterize growth patterns (from fetal life until the attainment of adult height) in patients with CAH on lifesaving GC treatment using the QEPS model growth functions and to compare patterns found between CAH groups of SW and SV, and with those seen in a healthy reference population, the GrowUp Gothenburg cohort (13).

## 2 Materials and methods

### 2.1 Study design

This was a retrospective, longitudinal, single-center cohort study conducted at Kaunas Clinics, Lithuania. Growth characteristics at birth and longitudinal growth patterns until adult height of a cohort of Lithuanian patients with classical CAH and a reference group of healthy Swedish individuals from the GrowUp_1974_Gothenburg cohort were compared (13, 15). Growth data were exported to the MATLAB software where estimations of the QEPS functions were made (14), **Figure 1**. The QEPS estimates were “incorporated” into the concept of different growth phases of a child, being early-life infancy, childhood, and pubertal (6).

### 2.2 Study population

#### 2.2.1 Patients with CAH

A total of 39 patients were diagnosed with CAH due to 21-hydroxylase deficiency between 1986 and 2008 at the Hospital of Lithuanian University of Health Science Kauno klinikos. Adequate longitudinal data from birth to adult height were available for 25 of these patients who comprised the study population: 12 boys (all with SW form) and 13 girls (8 with SW and 5 with SV form); CAH was diagnosed in newborn age in all patients in the study. The characteristics of the study population are given separately for boys and girls in **Tables 1a**, **b**. Patients with CAH were selected for the inclusion set on the following criteria: clinical diagnosis of classical CAH due to 21-hydroxylase deficiency, known length/weight and gestational age (GA) at birth, length/height measurements available from birth to adult height (measured by a Harpenden stadiometer at each visit), documented time of clinical signs of onset of puberty (as B2 or testicular volume ≥4 ml), data available on parent’s heights, and no evidence of other diseases or medications known to affect growth other than GCs. The absence of men with the SV form of CAH in our cohort is due to the retrospective nature of the study. All patients in the study were diagnosed clinically, men typically presenting with salt-wasting crises and women with ambiguous genitalia. In our population, men with simple virilizing CAH were typically diagnosed later in childhood and did not meet the inclusion criteria of having complete longitudinal growth data from infancy to adult height.

**Table 1a:** Subject characteristics at birth by sex compared with reference.

	Boys			Girls		
Variable	CAH N=12 mean±SD; median (Q1-Q3)	1974 reference N=1174	P-value	CAH N=13	1974 reference N=1165	P-value
GA (weeks)	39.9±1.4 40.5 (36.5-40.5)	40.2±1.1 40.5 (37.5-41.5)	0.71	39.6±3.2 40.5 (29.5-42.5)	40.3±1.0 40.5 (37.5-41.5)	0.83
BW (g)	3,616±556 3,660 (2,700-4,330)	3,517±486 3,520 (1,810-5,420)	0.48	3,238±768 3,360 (976-4150)	3,409±470 3,400 (1,620-5,670)	0.44
BW_SDS_	−0.24±0.91 −0.35 (−1.59-1.03)	−0.60±1.12 −0.50 (−6.67-2.58)	0.26	−0.71±1.18 −0.69 (−2.86-1.02)	−0.54±1.08 −0.49 (−5.48-3.55)	0.57
BL (cm)	53.2±2.8 53.0 (50.0-58.0)	50.5±2.1 51.0 (41.0-60.0)	<.0001	51.5±5.2 52.0 (37.0-58.0)	49.9±2.0 50.0 (42.0-58.0)	0.32
BL_SDS_	1.06±1.69 1.14 (−1.27-3.77)	0.05±1.01 0.01 (−3.52-3.14)	0.06	0.72±1.96 0.51 (−2.41-4.39)	0.01±1.00 0.00 (−3.47-2.65)	0.24
DiffMPH BL_SDS_	1.03±1.76 1.33 (−1.58-3.71)	0.06±0.84 0.06 (−2.30-3.15)	0.08	0.68±1.89 0.94 (−2.90-3.85)	0.05±0.81 0.05 (−3.07-3.07)	0.27
Mhei (cm)	166.9±2.4 167.0 (164.0-173.0)	166.8±6.2 167.0 (148.0-189.0)	0.87	166.6±6.1 168.0 (155.0-176.0)	166.1±5.9 166.0 (150.0-183.0)	0.77
Mhei_SDS_	0.02±0.39 0.03 (−0.45-1.01)	−0.00±1.00 0.03 (−3.05-3.60)	0.87	0.08±1.04 0.32 (−1.90-1.68)	0.00±1.00 −0.02 (−2.75-2.87)	0.77
Fhei (cm)	179.8±6.5 181.0 (170.0-192.0)	179.5±6.7 180.0 (160.0-202.0)	0.87	179.9±3.8 180.0 (174.0-186.0)	180.1±6.7 180.0 (158.0-208.0)	0.85
Fhei_SDS_	0.05±0.96 0.22 (−1.41-1.85)	0.00±1.00 0.07 (−2.89-3.33)	0.87	−0.03±0.57 −0.02 (-0.92-0.88)	0.00±1.00 −0.02 (−3.32-4.18)	0.85
TargetH_SDS_	−0.04±0.50 −0.03 (−0.90-0.76)	−0.07±0.71 −0.11 (−2.23-2.41)	0.89	−0.13±0.50 −0.15 (−1.20-0.51)	−0.15±0.64 −0.14 (−2.25-1.92)	0.90
MPH_SDS_	0.03±0.57 0.05 (−0.93-0.94)	0.00±0.81 −0.05 (−2.44-2.80)	0.89	0.03±0.63 −0.00 (−1.33-0.83)	−0.00±0.81 0.01 (−2.66-2.62)	0.90

BL, birth length; BW, birth weight; GA, gestation age; CAH, congenital adrenal hyperplasia; DiffMPH BL_SDS_, difference between birth length and mid-parental height; Fhei, father’s height; Mhei, mother’s height; SV, simple virilizing CAH form; SW, salt wasting CAH form; MPH, mid-parental height; SDS, standard deviation score; TargetH, target height.

Data are presented as mean±standard deviation, median (range), and number of observations, or number (percentage).

For test between two groups with respect to skewed continuous variables, Mann–Whitney U-test was used, and for normally distributed data, two-sample t-test was used.

**Table 1b:** Subject characteristics at birth for girls by type compared with reference.

Variable	Girls CAH-SW N=8	1974 reference N=1165	Girls CAH-SW vs. reference p-value	Girls CAH-SV N=5	1974 reference N=1165	Girls CAH-SV vs. reference p-value	Girls SW vs. SV p-value
GA (weeks)	40.4±0.8 40.5 (38.5-41.5)	40.3±1.0 40.5 (37.5-41.5)	0.85	38.3±5.1 40.5 (29.5-42.5)	40.3±1.0 40.5 (37.5-41.5)	0.56	0.63
BW (g)	3,473±384 3,370 (2,900-4150)	3,409±470 3,400 (1,620-5,670)	0.70	2,863±1,107 3,080 (976-3,800)	3,409±470 3,400 (1,620-5,670)	0.33	0.29
BW_SDS_	−0.40±0.97 −0.70 (−1.88-1.02)	−0.54±1.08 −0.49 (−5.48-3.55)	0.73	−1.19±1.45 −0.42 (−2.86-0.30)	−0.54±1.08 −0.49 (−5.48-3.55)	0.18	0.26
BL (cm)	53.9±2.4 54.0 (51.0-58.0)	49.9±2.0 50.0 (42.0-58.0)	<.0001	48.2±6.5 50.0 (37.0-53.0)	49.9±2.0 50.0 (42.0-58.0)	0.58	0.12
BL_SDS_	1.63±1.67 1.80 (−0.65-4.39)	0.01±1.00 0.00 (−3.47-2.65)	0.042	−0.57±1.70 0.30 (−2.41-1.15)	0.01±1.00 0.00 (−3.47-2.65)	0.49	0.049
DiffMPH BL_SDS_	1.42±1.48 1.65 (−0.32-3.85)	0.05±0.81 0.05 (−3.07-3.07)	0.049	−0.37±2.05 0.90 (−2.90-1.45)	0.05±0.81 0.05 (−3.07-3.07)	0.68	0.11
Mhei (cm)	166.9±7.3 168.5 (155.0-176.0)	166.1±5.9 166.0 (150.0-183.0)	0.73	166.2±4.4 166.0 (160.0-172.0)	166.1±5.9 166.0 (150.0-183.0)	0.98	0.86
Mhei_SDS_	0.13±1.24 0.40 (−1.90-1.68)	0.00±1.00 −0.02 (−2.75-2.87)	0.73	0.01±0.75 −0.02 (−1.05-1.00)	0.00±1.00 −0.02 (−2.75-2.87)	0.98	0.86
Fhei (cm)	181.5±3.6 182.5 (175.0-186.0)	180.1±6.7 180.0 (158.0-208.0)	0.56	177.4±2.8 178.0 (174.0-180.0)	180.1±6.7 180.0 (158.0-208.0)	0.36	0.055
Fhei_SDS_	0.21±0.54 0.36 (−0.77-0.88)	0.00±1.00 −0.02 (−3.32-4.18)	0.56	−0.41±0.42 −0.32 (−0.92 to −0.02)	0.00±1.00 −0.02 (−3.32-4.18)	0.36	0.055
TargetH_SDS_	−0.02±0.57 0.07 (−1.20-0.51)	−0.15±0.64 −0.14 (−2.25-1.92)	0.55	−0.30±0.35 −0.38 (−0.69-0.24)	−0.15±0.64 −0.14 (−2.25-1.92)	0.59	0.33
MPH_SDS_	0.17±0.72 0.28 (−1.33-0.83)	−0.00±0.81 0.01 (−2.66-2.62)	0.55	−0.20±0.44 −0.30 (−0.68-0.49)	−0.00±0.81 0.01 (−2.66-2.62)	0.59	0.33

AH, adult height; BL, birth length; BW, birth weight; GA, gestation age; CAH, congenital adrenal hyperplasia; DiffMPH BL_SDS_, difference between birth length and mid-parental height; Fhei, father’s height; Mhei, mother’s height; SV, simple virilizing CAH form; SW, salt wasting CAH form; MPH, mid-parental height; SDS, standard deviation score; TargetH, target height.

Data are presented as mean±standard deviation, median (range), and number of observations, or number (percentage).

For test between two groups with respect to skewed continuous variables, Mann–Whitney U-test was used and for normally distributed data two-sample t-test was used.

Although the initial diagnosis of classical CAH was based on clinical and biochemical criteria, genetic testing became available later in life for all patients. In all tested cases, genetic results confirmed the diagnosis of classical CAH, and genotype–phenotype concordance was observed. All included subjects were born before the introduction of newborn screening in 2015. Exclusion criteria included evidence of other chronic conditions or treatments known to affect growth, such as untreated celiac disease, chronic kidney disease, inflammatory bowel disease, congenital heart disease with chronic hypoxia, and growth hormone therapy. The initial cohort comprised all 39 patients with classical CAH treated at our center between 1986 and 2008. Of these, 25 patients were included in the final analysis due to sufficient longitudinal growth data, while whereas 14 were excluded because of missing or incomplete measurements (**Table 2**).

**Table 2:** Subject characteristics at birth by sex and included/excluded patients in the analysis.

	Boys			Girls		
Variable	Excluded CAH N=6	Included CAH N=12	P-value	Excluded CAH N=8	Included CAH N=13	P-value
GA (weeks)	40.2±0.8 40.5 (38.5-40.5)	39.9±1.4 40.5 (36.5-40.5)	0.94	40.1±0.7 40.5 (38.5-40.5)	39.6±3.2 40.5 (29.5-42.5)	0.70
BW (g)	3,362±574 3,650 (2,300-3,800)	3,616±556 3,660 (2,700-4,330)	0.38	3,596±530 3,603 (2,620-4,200)	3,238±768 3,360 (976-4,150)	0.26
BW_SDS_	−1.00±1.20 −0.38 (−3.12 to −0.05)	−0.24±0.91 −0.35 (−1.59-1.03)	0.15	−0.04±0.95 −0.13 (−1.70-1.12)	−0.71±1.18 −0.69 (−2.86-1.02)	0.19
BL (cm)	51.2±2.7 51.0 (47.0-55.0)	53.2±2.8 53.0 (50.0-58.0)	0.17	52.3±2.3 52.5 (48.0-55.0)	51.5±5.2 52.0 (37.0-58.0)	0.67
BL_SDS_	−0.36±1.42 −0.64 (−2.12-1.88)	1.06±1.69 1.14 (−1.27-3.77)	0.10	0.87±1.24 1.10 (−1.02-2.45)	0.72±1.96 0.51 (−2.41-4.39)	0.85
DiffMPH BL_SDS_	0.12±1.84 −0.10 (−2.29-2.54)	1.03±1.76 1.33 (−1.58-3.71)	0.33	1.14±0.72 1.33 (−0.49-1.79)	0.68±1.89 0.94 (−2.90-3.85)	0.45
Mhei (cm)	162.8±7.3 161.0 (153.0-174.0)	166.9±2.4 167.0 (164.0-173.0)	0.23	165.3±6.2 165.0 (156.0-176.0)	166.6±6.1 168.0 (155.0-176.0)	0.63
Mhei_SDS_	−0.64±1.18 −0.94 (−2.24-1.17)	0.02±0.39 0.03 (−0.45-1.01)	0.23	−0.15±1.06 −0.19 (−1.73-1.68)	0.08±1.04 0.32 (−1.90-1.68)	0.63
Fhei (cm)	177.3±2.0 177.5 (174.0-180.0)	179.8±6.5 181.0 (170.0-192.0)	0.24	177.5±7.7 177.5 (167.0-192.0)	179.9±3.8 180.0 (174.0-186.0)	0.43
Fhei_SDS_	−0.32±0.29 −0.30 (−0.82-0.07)	0.05±0.96 0.22 (−1.41-1.85)	0.24	−0.39±1.15 −0.39 (−1.97-1.78)	−0.03±0.57 −0.02 (−0.92-0.88)	0.43
TargetH_SDS_	−0.50±0.48 −0.52 (−1.23-0.08)	−0.04±0.50 −0.03 (−0.90-0.76)	0.08	−0.36±0.73 −0.38 (−1.61-0.55)	−0.13±0.50 −0.15 (−1.20-0.51)	0.39
MPH_SDS_	−0.48±0.54 −0.51 (−1.31-0.17)	0.03±0.57 0.05 (−0.93-0.94)	0.08	−0.27±0.93 −0.29 (−1.85-0.88)	0.03±0.63 −0.00 (−1.33-0.83)	0.39
Age at diagnosis (years)	1.4±2.1 0.1 (0.0-5.0)	0.1±0.1 0.1 (0.0-0.2)	0.30	2.5±4.8 0.0 (0.0-12.9)	1.8±2.9 0.1 (0.0-7.6)	0.64
Calendar year at diagnosis	2003±7 2005 (1994-2012)	1999±6 2001 (1986-2006)	0.15	2000±9 2004 (1984-2008)	2002±7 2005 (1994-2011)	0.64

AH, adult height; BL, birth length; BW, birth weight; GA, gestation age; CAH, congenital adrenal hyperplasia; DiffMPH BL_SDS_, difference between birth length and mid-parental height; Fhei, father’s height; Mhei, mother’s height; MPH, mid-parental height; SDS, standard deviation score; TargetH, target height.

Data are presented as mean±standard deviation, median (range), and number of observations, or number (percentage).

For test between two groups with respect to binary variables, Fisher’s exact test was used; for skewed continuous variables, Mann–Whitney U-test was used; and for normally distributed data, two-sample t-test was used.

##### 2.2.1.1 Replacement therapy

The effectiveness of the disease control was assessed based on the level of 17-hydroxy-progesterone (17-OHP), with the target range of 12–36 nmol/L (16), as well as clinical parameters of growth and bone age. All patients were treated with hydrocortisone (HC) until adult height (defined by growth velocity <2 cm/year and growth plates fusion confirmed by wrist X-ray). The infancy period was defined from birth to 1 year, childhood—from 1 year to onset of breast development in girls (Tanner stage B2) and testicular volume increase ≥ 4ml in boys (Tanner stage G2), and puberty period—from onset of puberty to adult height.

GC doses were expressed as median cumulative dose per body surface area (mg/m^2^/day) for the treatment period from diagnosis to the achieved final height. All GC doses used were converted to hydrocortisone (HC) dose equivalents (20 mg hydrocortisone = 5 mg prednisolone (PD) = 0.75 mg dexamethasone (DEX)) (17). In the total cohort, GC cumulative doses were 16.97 ± 4.73, 15.32 ± 3.0, and 15.55 ± 2.36 mg/m^2^/day, during infancy/childhood/puberty periods, respectively. Cumulative GC doses did not differ between the three groups of CAH patients with CAH-SW boys, CAH-SW girls, and CAH-SV girls during the entire growth period.

#### 2.2.2 Reference group from the GrowUp_1974_Gothenburg cohort

The reference group consisted of a subgroup of healthy individuals from the GrowUp_1974_Gothenburg cohort born in ~1974 in Sweden at full term (GA: 37−42 weeks) for whom birth characteristics and data on longitudinal growth until adult height were available (n = 2,339, boys 1,174, girls 1,165) (13, 15). Length/height and weight had been measured at well-baby clinics and child healthcare centers. A specially trained team measured the students at 17–18 years of age in their 11th school year using a calibrated Harpenden stadiometer. Additional measurements were made by the study team until adult height was achieved, as determined by when increase in height versus the previous year was 0.5cm or less. The study population and selection procedures have been described in detail elsewhere (14).

### 2.3 The QEPS growth model

The QEPS model was used to describe individual patterns of growth in terms of length/height from birth to adult height. Growth in height was modeled by a Quadratic (*Q*) function, a negative Exponential (*E*) function, a specific Pubertal (*P*) function, and a Stop (*S*) function (**Figure 1**; for details, see previous publications). The growth functions *E* and *P* were modified by both timescale and height-scale parameters, and *Q* and *S* modified by height-scale parameters, thus describing individualized growth with a shape-invariant model. The QEPS model separates total growth during puberty (*Tpubgain*) into two components; growth that is specific to puberty, *P-function* growth (*Pmax*, *Ppubgain*), and ongoing basic growth that is related to the *QES-functions* (*QESpubgain*). Details shown in **Figure 1**.

### 2.4 Definitions of growth variables used

For all QEPS variables (except *E_timescale_*), the height was given in cm and the individual’s age was given in weeks/months/years. The SDSs for the QEPS variables were computed from the reference population GrowUp_1990_Gothenburg.

#### 2.4.1 Basic total growth

The ongoing basic QE-growth, starting soon after conception, is described by *Q_max_, E_max_*, and *E_timescale_* for the prepubertal period, and as *QES-growth* after onset of puberty from when *S-function* is added.

#### 2.4.2 Early life growth period

*Fetal growth period* and *birth:* The fetal growth ends at birth (*QE_birth_*) and continues as postnatal infancy growth. Lithuanian CAH patients’ data (birth weight (BW), birth length (BL), and gestational age (GA)) were collected from medical records, with GA calculated from the last menstrual period. Also, for the birth-reference population, GA was estimated from the last menstrual period, with data originating from the Swedish medical birth registry (13) with birth length_SDS_ and weight_SDS_ derived from the 800,000 Swedish healthiest born 1990–1999 (14, 18). *Infancy growth period*: the infancy growth period (10) started at birth and ended at *Age_E99_*, i.e., the time when 99% of the *E-function’s* amplitude is reached, 1.9 years, as mean for the reference population, individually estimated. A shorter E-timescale means that the Emax is reached faster, at a younger age than with a longer E-timescale; a higher E-heightscale means that the height of the infant/child due to the E-function is higher than for an infant/child with lower E-heightscale.

#### 2.4.3 Childhood growth period

Tempo describes childhood duration, starting from *Age_E99_*, ending at the age at onset of pubertal growth, *Age_P5_*, whereas the childhood growth was calculated vs. *Q_max_, E_max_* and *QE_max_*, since the correlation between *QE_maxSDS_* and QE*_AgeP5SDS_* is 0.993 for women and 0.996 for men.

#### 2.4.4 Pubertal growth period

*Pubertal growth.* During puberty, the QEPS growth model can separate the specific pubertal *P-function* growth, *P_max_*, or as *Ppubgain*(*P_AgeP5-P100_*), from the ongoing basic *QES-functions* (*QES_AgeP5-P100_)* of growth and thus describe the total gain (*T_AgeP5-P100_)* in height during puberty (13), also the *Deltapubgain*, i.e., *Ppubgain* – *QESpubgain* was calculated.

*Timing and tempo.* Here, we primarily defined the onset of pubertal growth period (13) when 5% of *P*-function growth was attained (*AgeP_5_*) for mid with *AgeP_50_*, end of pubertal growth as the age at which 95% of *P*-*function* growth had been completed (*AgeP_95_*). *Tonset, age at PHV* (13, 14) *(Age_TPHV_)*, and *Tend* were additionally calculated from the *Total* growth curve.

#### 2.4.5 Total growth (T_max_)

the sum of *E, Q*, and *P* minus *S*. The *S*-function is stopping the *Q*-*function* growth, assumed to end when the curve reaches its maximum amplitude (*T_max_*).

#### 2.4.6 Adult height

In the reference subgroup of GrowUp_1974_Gothenburg here used, the AH_SDS_ used in this study originated from the reference population, i.e., the 2,339, boys 1,174 and girls 1,165, a subgroup of GrowUp_1974_Gothenburg (13, 15). DiffSDS: The calculated difference between the individual’s actual, at a timepoint/age, height_SDS_ and the individual’s mid-parental height SDS (MPH_SDS_) diffH-MPH_SDS_, the intrafamilial height difference.

#### 2.4.7 Parental heights

*For the CAH patients:* Data on parental heights were mainly obtained by measurement in the outpatient clinic, but in some, parental height was self-reported. MPH_SDS_ was calculated vs. the reference population here used. *For the reference population:* The mother heights of our study participants originated from the Swedish medical birth registry (14), whereas the father heights came from the questionnaires answered by the parents or the study participants in the high schools, or from records at child healthcare centers. The parental height_SDS_ were derived from the current study population, by computing the SDS based on the heights of all 1,174 fathers and 1,165 mothers.

### 2.5 Statistical analysis

Continuous variables were described by mean, standard deviation, median, minimum, and maximum, and categorical by counts and percentages.

For test between two groups, Fisher’s exact test was used for dichotomous, Mann–Whitney U-test for skewed continuous variables and two-sample t-test for normally distributed continuous variables. The standardized mean difference was described as an effect size for comparison of CAH vs. 1974 reference considering QEPS variables.

All tests were two-tailed. Results were interpreted at the 0.05 significance level. All analyses were performed by using SAS^®^ Software v9.4 (SAS Institute Inc., Cary, NC, USA).

### 2.6 Ethical approval

The materials in this study were collected from databases containing longitudinal measurements from individuals that had already been approved for scientific use. There was no risk of discomfort or harm to the participants. Data from patients with CAH were collected at the Endocrinology Department of the Hospital of the Lithuanian University of Health Sciences, Kauno klinikos, after obtaining permission from the Kaunas Regional Committee for Biomedical Ethics (No. BE-2-29, approved on 23 April 2018). All participants and/or their guardians had provided informed written consent for collecting retrospective data from medical records and subsequent use of these data for analysis.

For subjects in the GrowUp_1974_Gothenburg cohort, ethical approval was obtained from the Regional Ethics Review Board in Gothenburg (Ad 91-92/131–93 and 444–08 T062-09), and all participants gave informed consent (informed consent from legal guardians was given for individuals <18 years of age).

## 3 Results

### 3.1 Description of the CAH study population

**Figure 2:**
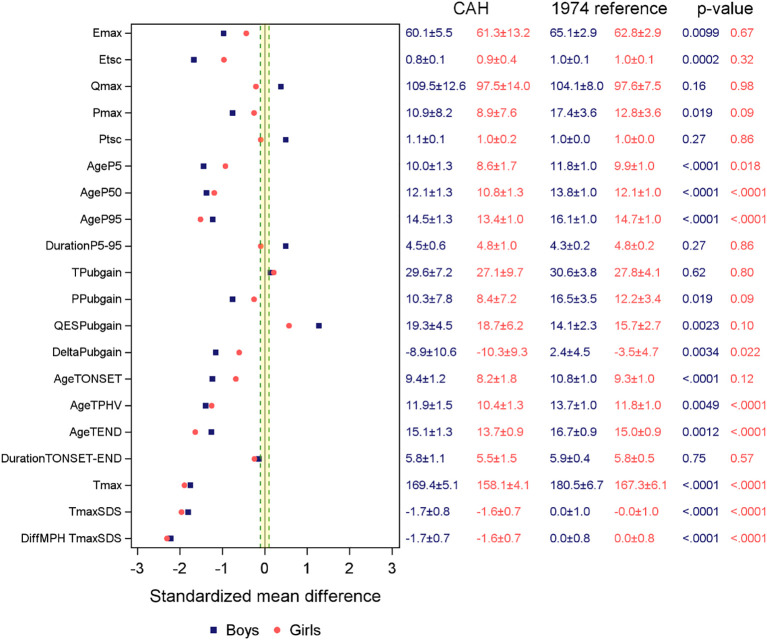
Standardized mean difference for QEPS variables by sex and cohort. Abbreviations: CAH, congenital adrenal hyperplasia; AgeP5, age at which 5% of the P-function growth is reached**;** AgeP_50_, age at which 50% of the P-function growth is reached**;** AgeP_95_, age at which 95% of the P-function growth is reached**;** Age_TEND_, age at the end of puberty where the height velocity has decreased to 1 cm/year for function T’(age); Age_TONSET_, age (years) at minimum height velocity of the T-function at start of the pubertal growth**;** AgeT_PHV_, age at peak height velocity of the T-function; Duration P_P5-95_, duration (years) between AgeP_5_ and AgeP_95_; Duration T_ONSET-END_, duration between T_ONSET-and_ T_END_; *E_max_*, gain in adult height in cm due to *E-function* growth; E_tsc_, individual time (years) scale ratio, modifying the time scale of the E-function growth; T_max_, modeled total adult height in cm; T_max_, Q_max_ + E_max_ + P_max_ − S_max_; T_max_SDS, standard deviation score for T_max_; DiffMPH T_max_SDS, difference between T_max_ and mid-parental height; Deltapubgain, Ppubgain minus QESpubgain; E_max_, gain in adult height in cm due to E-function growth; E_tsc_, individual time (years) scale ratio, modifying the time scale of the E-function growth; Q_max_, gain in adult height in cm due to Q-function growth; P_max_, pubertal gain in adult height in cm due to the P-function growth; P_tsc_, individual time (years) scale ratio in the P-function growth.

**Figure 3:**
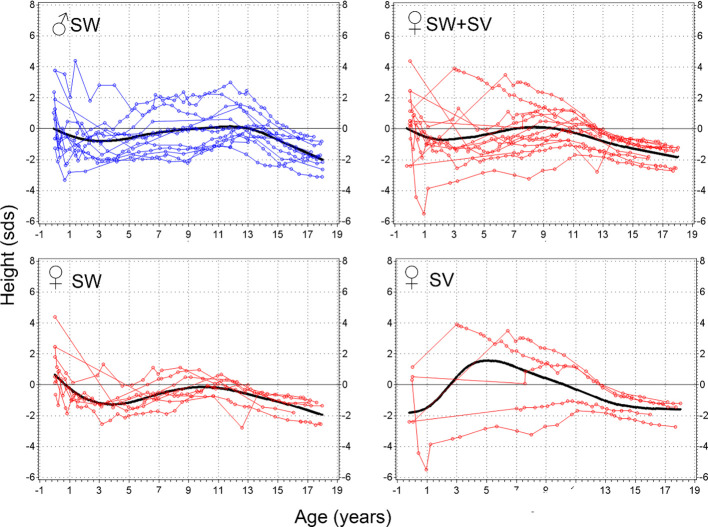
Horizontal growth Standard Deviation Scores (SDS) from birth to adult height. These figures illustrate the standardized growth deviation trajectories (SDS) for patients with CAH compared with the healthy reference cohort from birth to adult height. Boys and girls with CAH show distinct growth patterns, including differences in pubertal timing and total height gain. Abbreviations: CAH, congenital adrenal hyperplasia; SDS, standard deviation score; SV, simple virilizing CAH form; SW, salt wasting CAH form.

**Figure 4:**
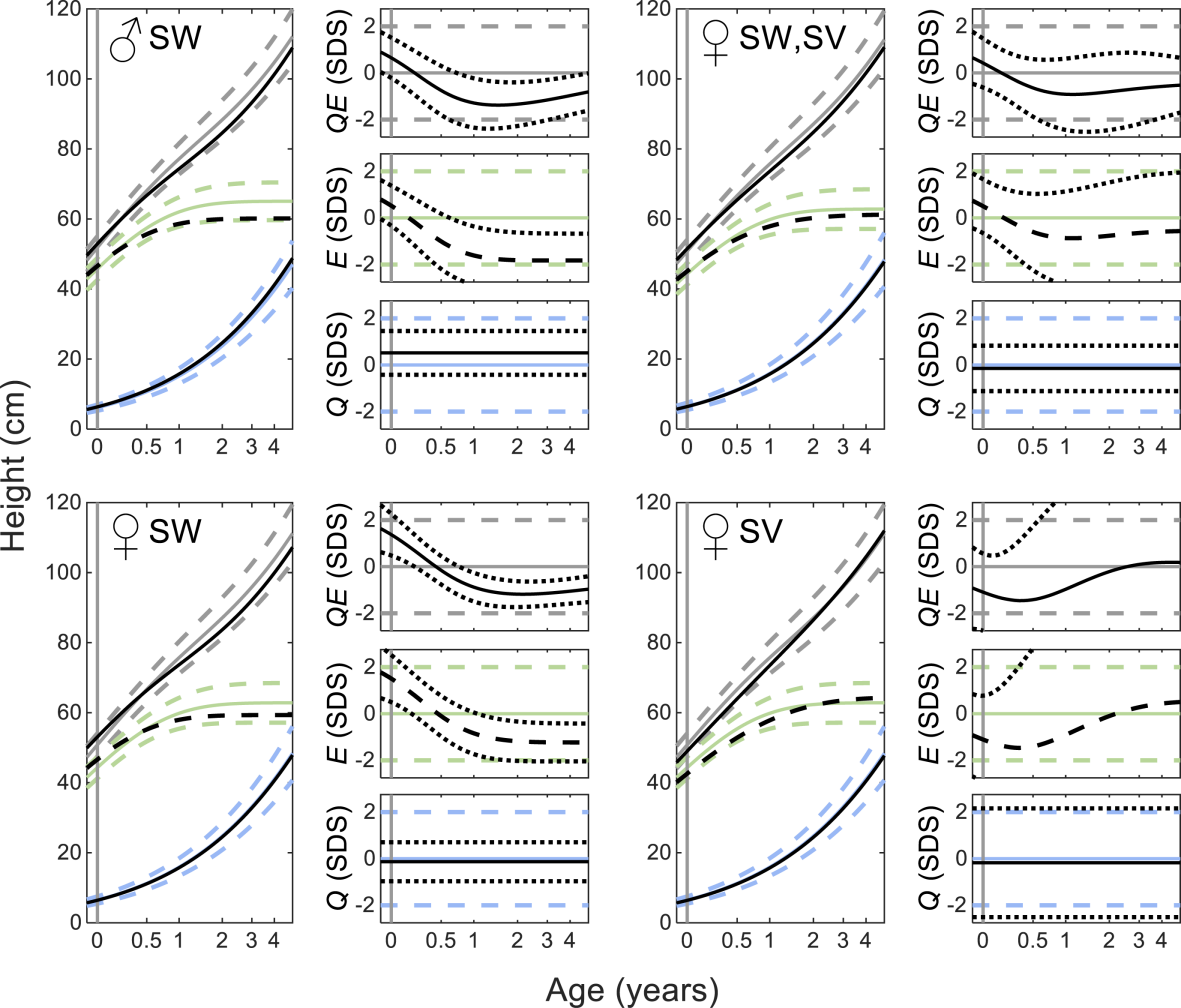
Growth trajectories (length/height in cm) from birth to 4 years of age. Growth patterns during early childhood (birth to 4 years) in CAH patients compared with the reference population. The figure highlights the reduced early growth in CAH-SW children, with emphasis on differences in height gain driven by the QEPS model’s early-life growth (E-function). Abbreviations: CAH, congenital adrenal hyperplasia; SV, simple virilizing CAH form; SW, salt wasting CAH form.

**Figure 5:**
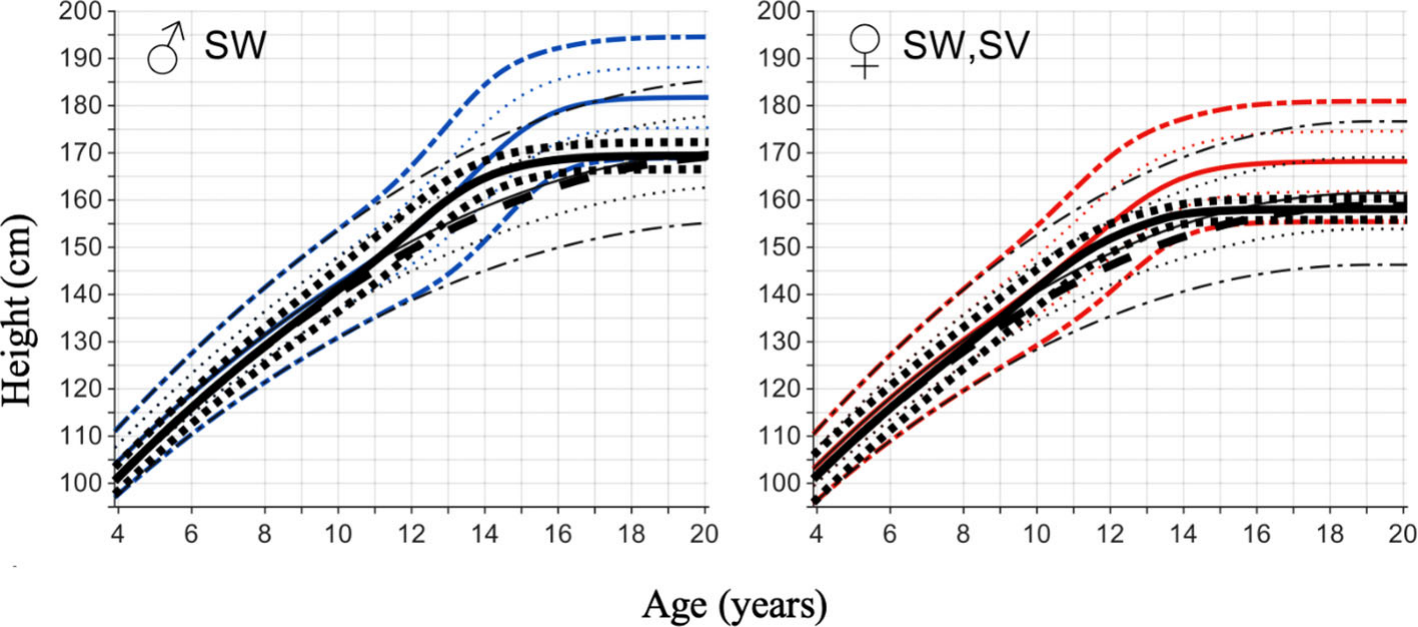
Longitudinal growth trajectories from 4 years to adult height. Longitudinal growth from mid-childhood (4 years) to adult height for patients with CAH versus the reference cohort. The chart demonstrates the impact of basic ongoing growth (Q-function) and puberty-specific growth (P-function) on the total height outcomes. Abbreviations: SV, simple virilizing CAH form; SW, salt wasting CAH form.

**Figure 6:**
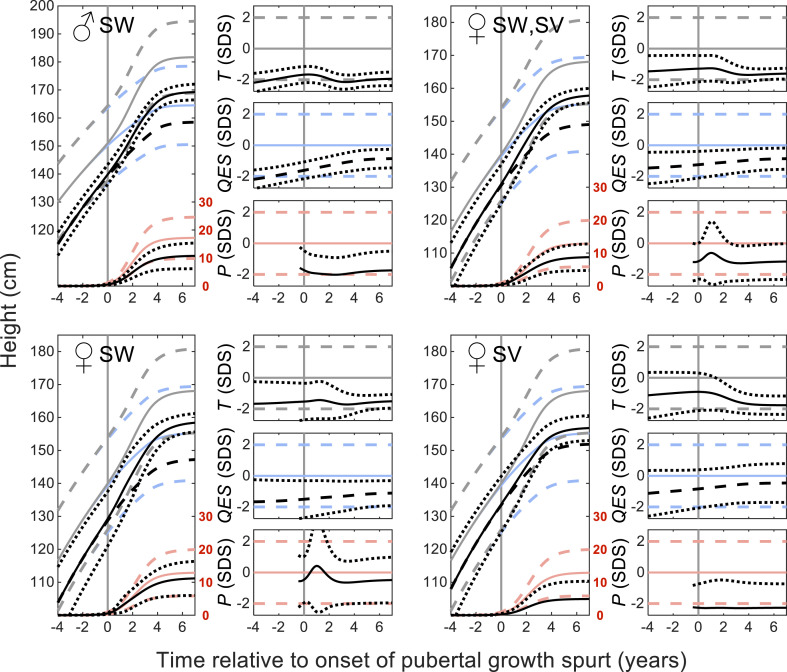
Mean growth trajectories for CAH patients aligned with pubertal age (P-Age) references. This figure compares the mean growth trajectories of CAH patients with the reference population, aligned according to pubertal age (P-age). It demonstrates the total growth patterns (QEPS-function) as well as the separation of growth components into puberty-specific (P-function) and independent (QES-function) contributions. The alignment allows for a clearer analysis of pubertal timing and growth tempo differences between the CAH and reference groups. Abbreviations: SV, simple virilizing CAH form; SW, salt wasting CAH form.

**Figure 7:**
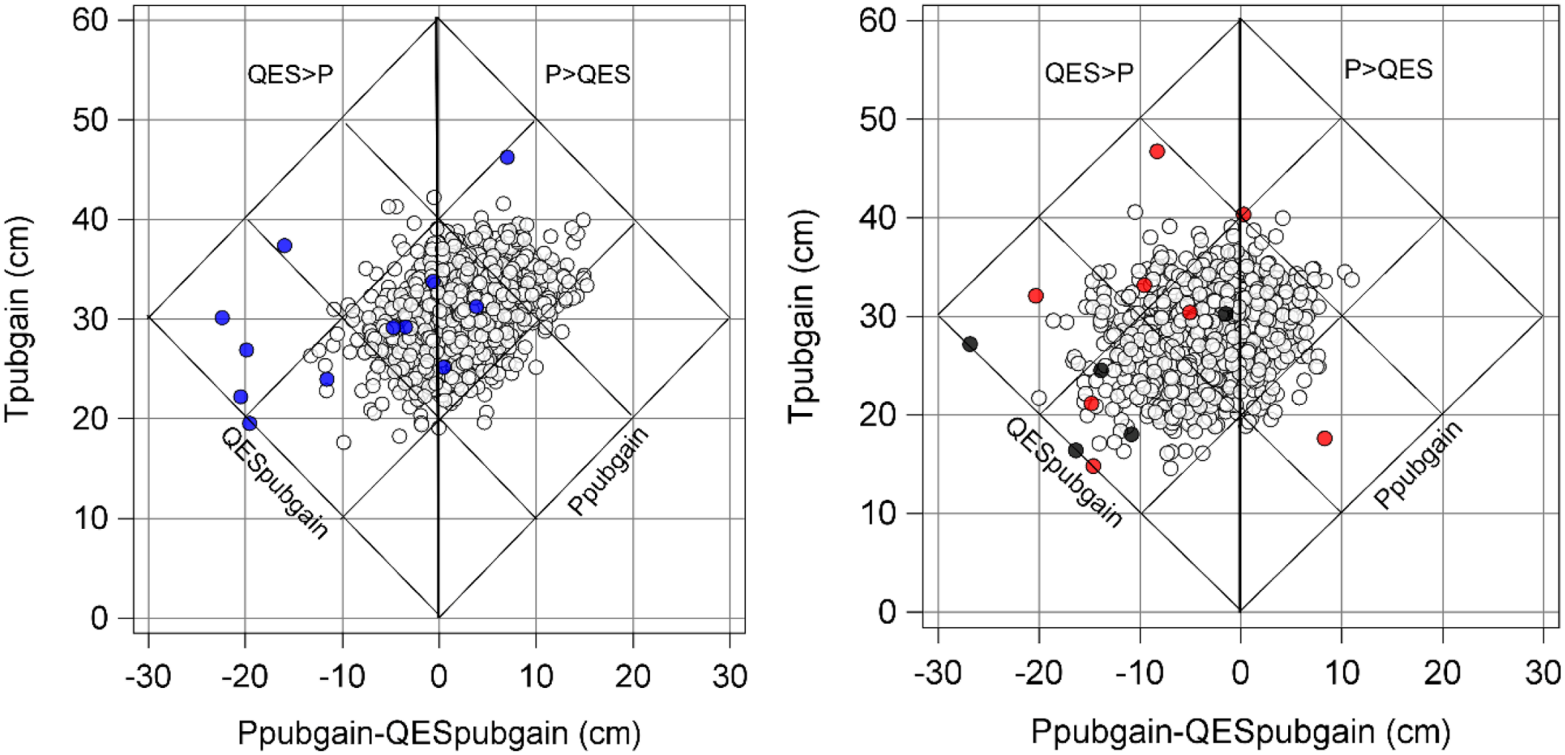
Growth during puberty. Left: Relationship between growth in centimeters for specific pubertal growth, Ppubgain (*P_AgeP5-P100_)*, and gain in height due to pubertal growth of the *QES* function, QESpubgain (*QES_AgeP5-P100_)*, in boys with salt loosing CAH. On the y-axis, the total growth during puberty Tpubgain (*T_AgeP5-P100_)* is given, and on the x-axis, the difference between Ppubgain and QESpubgain. The bold vertical reference at x=0 line indicates where Ppubgain=QESpubgain. Combinations for Ppubgain and QESpubgain can be read on the oblique axes. Unfilled circles indicate corresponding values for boys in the reference population for height in Swedish boys born around 1974. Blue filled circles show boys with CAH. Right: Corresponding figure for girls with CAH. Red symbols indicate girls with salt loosing syndrome and black symbol girls with simple virilizing CAH. Unfilled circles indicate corresponding reference for Swedish girls.

Baseline characteristics for the study population of 25 patients, 12 boys (SW) and 13 girls (8 SW, 5 SV), with CAH and the reference cohort of 1,174 boys and 1,165 girls from the GrowUp_1974_Gothenburg study are shown in **Table 1a** for CAH-SW boys and all girls, and in **Table 1b** for the girls divided into CAH-SW and CAH-SV. Comparison of the QEPS-model estimations for both groups is shown in **Table 3** and **Figure 2**, the Forest plot, length/height vs. horizontal SDS in **Figure 3**, vs. C-age (14) in **Figure 4** birth–4 years, vs. C-age in **Figure 5**–4 years to adult height, vs. P-age ref in **Figure 6**, and total growth, *Tpubgain*, divided during puberty into *Ppubgain* and *QESpubgain* for *Deltapubgain*, i.e., *Ppubgain* – *QESpubgain* in **Figure 7**.

**Table 3a:** QEPS variables by sex compared with reference.

	Boys			Girls		
Variable	CAH N=12	1974 reference N=1174	P-value	CAH N=13	1974 reference N=1165	P-value
E_max_	60.1±5.5 58.9 (52.0-72.3)	65.1±2.9 65.1 (56.6-74.8)	0.0099	61.3±13.2 58.6 (47.4-91.4)	62.8±2.9 62.9 (52.5-73.3)	0.67
E_tsc_	0.8±0.1 0.8 (0.5-1.0)	1.0±0.1 1.0 (0.7-1.3)	0.0002	0.9±0.4 0.8 (0.5-2.0)	1.0±0.1 1.0 (0.7-1.3)	0.32
Q_max_	109.5±12.6 107.0 (86.5-135.4)	104.1±8.0 103.9 (73.7-135.3)	0.16	97.5±14.0 99.2 (77.7-127.0)	97.6±7.5 97.6 (75.1-125.1)	0.98
P_max_	10.9±8.2 12.1 (0.0-28.0)	17.4±3.6 17.5 (4.1-28.9)	0.019	8.9±7.6 6.2 (0.0-21.4)	12.8±3.6 12.7 (0.9-23.6)	0.09
P_tsc_	1.1±0.1 1.0 (0.8-1.4)	1.0±0.0 1.0 (0.8-1.2)	0.27	1.0±0.2 1.0 (0.4-1.4)	1.0±0.0 1.0 (0.8-1.2)	0.86
AgeP_5_	10.0±1.3 9.9 (8.1-12.8)	11.8±1.0 11.8 (8.6-14.9)	<.0001	8.6±1.7 8.8 (6.3-12.4)	9.9±1.0 9.8 (7.2-13.0)	0.018
AgeP_50_	12.1±1.3 12.0 (10.3-15.0)	13.8±1.0 13.8 (10.7-16.9)	<.0001	10.8±1.3 11.0 (9.0-13.2)	12.1±1.0 12.1 (9.6-15.4)	<.0001
AgeP_95_	14.5±1.3 14.2 (12.8-17.5)	16.1±1.0 16.1 (13.1-19.3)	<.0001	13.4±1.0 13.4 (11.6-15.6)	14.7±1.0 14.7 (12.2-18.1)	<.0001
Duration P_P5-95_	4.5±0.6 4.4 (3.6-6.2)	4.3±0.2 4.3 (3.2-5.0)	0.27	4.8±1.0 4.9 (1.9-6.5)	4.8±0.2 4.8 (3.8-5.5)	0.86
T_pubgain_	29.6±7.2 29.2 (19.5-46.2)	30.6±3.8 30.6 (17.6-42.2)	0.62	27.1±9.7 27.2 (14.8-46.7)	27.8±4.1 27.8 (14.6-40.6)	0.80
P_pubgain_	10.3±7.8 11.5 (0.0-26.6)	16.5±3.5 16.6 (3.9-27.4)	0.019	8.4±7.2 5.9 (0.0-20.3)	12.2±3.4 12.1 (0.9-22.4)	0.09
QES_pubgain_	19.3±4.5 18.7 (12.3-26.7)	14.1±2.3 14.1 (7.5-23.2)	0.0023	18.7±6.2 18.0 (4.7-27.5)	15.7±2.7 15.5 (8.0-25.5)	0.10
Delta_pubgain_	−8.9±10.6 −8.1 (-22.4-7.0)	2.4±4.5 2.3 (−13.3-15.2)	0.0034	−10.3±9.3 −10.8 (−26.8-8.3)	-3.5±4.7 -3.5 (-20.0-11.0)	0.022
AgeT_ONSET_	9.4±1.2 9.4 (7.7-12.0)	10.8±1.0 10.7 (7.5-13.9)	<.0001	8.2±1.8 8.3 (5.6-11.5)	9.3±1.0 9.2 (6.3-12.6)	0.12
AgeT_PHV_	11.9±1.5 12.1 (9.7-14.8)	13.7±1.0 13.7 (10.6-16.8)	0.0049	10.4±1.3 10.4 (8.5-13.2)	11.8±1.0 11.8 (9.4-15.2)	<.0001
AgeT_END_	15.1±1.3 14.8 (13.4-17.8)	16.7±0.9 16.7 (14.0-19.6)	0.0012	13.7±0.9 13.7 (12.2-15.1)	15.0±0.9 15.0 (12.9-18.2)	<.0001
Duration T_ONSET-END_	5.8±1.1 5.8 (4.7-8.5)	5.9±0.4 5.9 (4.4-7.2)	0.75	5.5±1.5 5.6 (3.1-8.1)	5.8±0.5 5.8 (3.8-7.1)	0.57
T_max_	169.4±5.1 169.0 (159.6-176.2)	180.5±6.7 180.3 (157.3-201.1)	<.0001	158.1±4.1 159.2 (150.7-163.7)	167.3±6.1 167.2 (145.7-183.4)	<.0001
T_max_SDS	−1.7±0.8 −1.8 (−3.2 to −0.7)	0.0±1.0 −0.0 (-3.5-3.0)	<.0001	−1.6±0.7 −1.4 (−2.8 to −0.6)	−0.0±1.0 −0.0 (−3.5-2.6)	<.0001
DiffMPH T_max_SDS	−1.7±0.7 −1.7 (−2.9 to −0.7)	0.0±0.8 0.0 (−2.3-3.1)	<.0001	−1.6±0.7 −1.4 (−3.0 to −0.7)	0.0±0.8 0.0 (−3.1-3.1)	<.0001

AgeP5, age at which 5% of the P-function growth is reached**;** AgeP_50_, age at which 50% of the P-function growth is reached**;** AgeP_95_, age at which 95% of the P-function growth is reached**;** Age_TEND_, age at the end of puberty where the height velocity has decreased to 1 cm/year for function T’(age); Age_TONSET_, age at minimum height velocity of the T-function at start of the pubertal growth**;** AgeT_PHV_, age at peak height velocity of the T-function; Duration P_P5-95_, duration between AgeP_5_ and AgeP_95_; Duration T_ONSET-END_, duration between T_ONSET-and_ T_END_; T_max_, modeled total adult height in cm; T_max_, Q_max_ + E_max_ + P_max_ − S_max_; T_max_SDS, standard deviation score for T_max_; DiffMPH T_max_SDS, difference between T_max_ and mid-parental height; E_max_, gain in adult height in cm due to E-function growth,.

E_tsc_, individual time scale ratio, modifying the time scale of the E-function growth; Q_max_, gain in adult height in cm due to Q-function growth; P_max_, pubertal gain in adult height in cm due to the P-function growth; P_tsc_, individual time scale ratio in the P-function growth.

Data are presented as mean±standard deviation, median (range), and number of observations, or number (percentage).

For test between two groups with respect to normally distributed data, two-sample t-test was used.

**Table 3b:** QEPS variables for girls by type compared with reference.

							Girls SW vs. SV
Variable	Girls CAH-SW N=8	1974 Reference N=1165	Girls CAH-SW vs. reference p-value	Girls CAH-SV N=5	1974 Reference N=1165	Girls CAH-SV vs. reference p-value	Girls SW vs. SV p-value
E_max_	59.3±3.3 59.8 (55.1-64.3)	62.8±2.9 62.9 (52.5-73.3)	0.0006	64.4±22.0 50.3 (47.4-91.4)	62.8±2.9 62.9 (52.5-73.3)	0.89	0.64
E_tsc_	0.8±0.1 0.8 (0.5-0.9)	1.0±0.1 1.0 (0.7-1.3)	<.0001	1.1±0.6 0.8 (0.5-2.0)	1.0±0.1 1.0 (0.7-1.3)	0.85	0.38
Q_max_	97.6±9.5 100.4 (82.8-110.8)	97.6±7.5 97.6 (75.1-125.1)	0.99	97.3±20.9 93.9 (77.7-127.0)	97.6±7.5 97.6 (75.1-125.1)	0.98	0.97
P_max_	11.3±7.6 12.9 (0.1-21.4)	12.8±3.6 12.7 (0.9-23.6)	0.60	4.9±6.2 3.8 (0.0-15.1)	12.8±3.6 12.7 (0.9-23.6)	0.047	0.14
P_tsc_	1.0±0.3 1.0 (0.4-1.4)	1.0±0.0 1.0 (0.8-1.2)	0.90	1.0±0.0 1.0 (0.9-1.0)	1.0±0.0 1.0 (0.8-1.2)	0.63	0.98
AgeP_5_	8.7±2.1 8.4 (6.3-12.4)	9.9±1.0 9.8 (7.2-13.0)	0.15	8.5±0.8 8.8 (7.7-9.3)	9.9±1.0 9.8 (7.2-13.0)	0.0021	0.87
AgeP_50_	10.9±1.6 10.7 (9.0-13.2)	12.1±1.0 12.1 (9.6-15.4)	0.07	10.7±0.7 11.0 (9.9-11.6)	12.1±1.0 12.1 (9.6-15.4)	0.0015	0.84
AgeP_95_	13.4±1.2 13.3 (11.6-15.6)	14.7±1.0 14.7 (12.2-18.1)	0.0003	13.3±0.7 13.4 (12.5-14.3)	14.7±1.0 14.7 (12.2-18.1)	0.0012	0.81
Duration P_P5-95_	4.8±1.3 4.9 (1.9-6.5)	4.8±0.2 4.8 (3.8-5.5)	0.90	4.8±0.2 4.9 (4.4-5.0)	4.8±0.2 4.8 (3.8-5.5)	0.63	0.98
T_pubgain_	29.5±11.1 31.2 (14.8-46.7)	27.8±4.1 27.8 (14.6-40.6)	0.68	23.3±5.9 24.5 (16.4-30.2)	27.8±4.1 27.8 (14.6-40.6)	0.013	0.27
P_pubgain_	10.8±7.2 12.2 (0.1-20.3)	12.2±3.4 12.1 (0.9-22.4)	0.60	4.7±5.9 3.6 (0.0-14.4)	12.2±3.4 12.1 (0.9-22.4)	0.047	0.14
QES_pubgain_	18.8±7.1 19.0 (4.7-27.5)	15.7±2.7 15.5 (8.0-25.5)	0.26	18.6±5.0 16.4 (14.4-27.0)	15.7±2.7 15.5 (8.0-25.5)	0.26	0.96
Delta_pubgain_	−8.0±9.2 −8.9 (−20.3-8.3)	−3.5±4.7 −3.5 (−20.0-11.0)	0.21	−13.9±9.2 −13.8 (−26.8 to −1.5)	−3.5±4.7 −3.5 (−20.0-11.0)	0.06	0.29
AgeT_ONSET_	7.9±2.1 7.3 (5.6-11.5)	9.3±1.0 9.2 (6.3-12.6)	0.16	8.9±0.7 9.0 (8.2-9.6)	9.3±1.0 9.2 (6.3-12.6)	0.59	0.47
AgeT_PHV_	10.2±1.6 9.9 (8.5-13.2)	11.8±1.0 11.8 (9.4-15.2)	0.035	10.8±0.3 10.9 (10.4-11.1)	11.8±1.0 11.8 (9.4-15.2)	0.06	0.56
AgeT_END_	13.8±0.9 13.8 (12.2-15.1)	15.0±0.9 15.0 (12.9-18.2)	<.0001	13.4±0.8 13.4 (12.4-14.5)	15.0±0.9 15.0 (12.9-18.2)	<.0001	0.47
Duration T_ONSET-END_	5.7±1.7 6.0 (3.1-8.1)	5.8±0.5 5.8 (3.8-7.1)	0.91	5.0±0.7 4.9 (4.4-5.8)	5.8±0.5 5.8 (3.8-7.1)	0.0096	0.55
T_max_	158.8±4.1 159.7 (152.4-163.7)	167.3±6.1 167.2 (145.7-183.4)	<.0001	156.9±4.3 159.0 (150.7-160.9)	167.3±6.1 167.2 (145.7-183.4)	0.0001	0.45
T_max_SDS	−1.5±0.7 −1.3 (−2.5 to −0.6)	−0.0±1.0 −0.0 (−3.5-2.6)	<.0001	−1.8±0.7 −1.4 (−2.8 to −1.1)	−0.0±1.0 −0.0 (−3.5-2.6)	<.0001	0.45
DiffMPH T_max_SDS	−1.6±0.8 −1.5 (−3.0 to −0.7)	0.0±0.8 0.0 (−3.1-3.1)	<.0001	−1.6±0.8 −1.2 (−2.7 to −0.8)	0.0±0.8 0.0 (−3.1-3.1)	<.0001	0.90

AgeP5, age at which 5% of the P-function growth is reached**;** AgeP_50_, age at which 50% of the P-function growth is reached**;** AgeP_95_, age at which 95% of the P-function growth is reached**;** Age_TEND_, age at the end of puberty where the height velocity has decreased to 1 cm/year for function T’(age); Age_TONSET_, age at minimum height velocity of the T-function at start of the pubertal growth**;** AgeT_PHV_, age at peak height velocity of the T-function; Duration P_P5-95_, duration between AgeP_5_ and AgeP_95_; Duration T_ONSET-END_, duration between T_ONSET-and_ T_END_; *E_max_*, gain in adult height in cm due to *E-function* growth; E_tsc_, individual time scale ratio, modifying the time scale of the E-function growth; T_max_, modeled total adult height in cm; T_max_, Q_max_ + E_max_ + P_max_ − S_max_; T_max_SDS, standard deviation score for T_max_; DiffMPH T_max_SDS, difference between T_max_ and mid-parental height; Deltapubgain, Ppubgain minus QESpubgain; E_max_, gain in adult height in cm due to E-function growth; E_tsc_, individual time scale ratio, modifying the time scale of the E-function growth; Q_max_, gain in adult height in cm due to Q-function growth; P_max_, pubertal gain in adult height in cm due to the P-function growth; P_tsc_, individual time scale ratio in the P-function growth.

Data are presented as mean±standard deviation, median (range), and number of observations, or number (percentage).

For test between two groups with respect to normally distributed data, two-sample t-test was used.

### 3.2 Early life growth period

Except for length at birth for boys and girls with SW, birth characteristics (BW, BL, and GA) and parental heights were similar for children in the CAH and GrowUp1974 cohorts. Boys with CAH-SW were significantly longer at birth than boys in the reference population (53.2 ± 2.8 vs. 50.5 ± 2.1 cm, p<0.0001), but of similar birth length compared with Lithuanian newborn reference (53.2 ± 2.8 vs. 53.0 ± 2.1 cm, p >0.05) (**Table 1a**). A subsequent analysis showed that girls with CAH-SW were also significantly longer at birth than girls in the reference population (**Table 1b**), but similar with Lithuanian newborn reference (53.9 ± 2.4vs. 52.3 ± 2.1 cm, p>0.05) (19), whereas girls with CAH-SV did not differ in birth length from neither reference.

The QEPS model revealed that both boys and girls with CAH-SW had a faster fetal–infancy growth rate as evidenced by a significantly shorter *E-timescale* (the E-growth function in the QEPS-model representing mainly early life growth) compared with the reference population (p=0.0002 and p<0.0001, respectively). The amplitude/magnitude of this specific fetal–infancy growth function was lower in both boys and girls with CAH-SW, lower *Emax* (p=0.0099 and p = 0.0006, respectively) (**Table 1a**, **b**; **Figure 2**). No significant differences were found for girls with CAH-SV.

### 3.3 Childhood growth period

Childhood growth, which is represented by the *Q-function* of the QEPS growth model, was similar prior to puberty for boys and girls in the CAH cohort relative to their peers in the reference cohort (**Table 3**; **Figures 2**, **3**).

### 3.4 Pubertal growth period

*The tempo of pubertal growth* differed significantly between the groups. Both boys and girls with CAH were significantly younger than the reference group at the onset (AgeP5), mid (AgeP50, PPHV), and end (AgeP95) of puberty; however, the duration of puberty was the same for both boys and girls versus their references. Specific pubertal growth for children with CAH began on average 1.85 years earlier in boys and 1.27 years earlier in girls and ended 1.77 years earlier in boys and 1.45 years earlier in girls compared with the reference cohort. Mean age at PHV for the CAH/reference cohorts was 10.4/11.8 years for girls (p<0.0001) and 11.9/13.7 years for boys (p=0.0049) (**Table 3**; **Figure 2**, **3**).

*Total growth during puberty* (Tpubgain) was similar in boys and SW girls in the CAH and reference cohorts whereas the specific pubertal growth was lower in CAH-SV girls (**Tables 3a, b**; **Figure 2**). However, the growth pattern for boys with CAH differed significantly from that of boys in the reference cohort: More of the growth in the former cohort was attributed to the continuation of ongoing basic growth (QESpubgain; p=0.0023), and less was specific to puberty (Ppubgain; p=0.019) than in their counterparts. Thus, the median of total height gain that was specific to puberty (Pmax) was lower at 5.4cm in boys with CAH (p=0.019). A similar overall pattern was observed in CAH-SV girls, and the Tpubgain was lower, due to less Ppubgain and lower Pmax (**Tables 3a, b**; **Figures 2**, **7**). The Deltapubgain became shifted to the left in CAH patients compared with the reference (**Figure 7**) mainly due to the earlier onset of puberty.

Changes in height of patients with CAH are visualized in **Figure 5** according to sex versus the growth references vs. P-age, which shows total growth (QEPS-function related) and growth separated out into the components that are specific (P-function-related) and independent (QES-function-related) of puberty. Data for each sex have been aligned with the reference according to age at the onset of puberty, P-age, to allow comparison.

Patients with the CAH-SV form demonstrated earlier pubertal onset and reduced pubertal height gain compared with CAH-SW patients. Patients with both CAH forms exhibited reduced adult height compared with population references, with more pronounced impairment observed in CAH-SW patients.

### 3.5 Adult height

Young men and women with CAH were shorter at adult height than the reference group by an average of 11.1 and 9.2cm, respectively; AH_SDS_ was −1.7 (0.8) and −1.6 (0.7), respectively (**Tables 1a, b**; **Figures 2**–**6**). The adult height achieved in patients with CAH was also significantly below their MPH, target height, by on average 8.65cm in girls and 10.42cm in boys, with a diffSDS of −1.7 and −1.6, respectively (both p<0.0001), and when divided, AH_SDS_ in SWgirls −1.5, SVgirls −1.8, and diffSDS −1.6 in boys (**Tables 3a, b**; **Figure 2**).

## 4 Discussion

### 4.1 Principal findings

Longitudinal growth is an integrated marker of health and an important clinical outcome in children and adolescents with CAH receiving lifelong GC replacement therapy. To our knowledge, this is the first study to characterize growth patterns from birth to adult height in patients with CAH using the growth model. We analyzed birth characteristics and longitudinal growth across distinct growth phases—early life (fetal, birth, and infancy), childhood, and puberty (6)—and compared these patterns with those of a healthy reference population (13, 20). The QEPS model enabled separation of these growth phases and proved useful for characterizing length/height trajectories in patients with CAH.

Children with CAH had distinctly different growth patterns when compared with a healthy reference population. Both boys and girls with the SW form of CAH were longer at birth than their peers in the reference populations and showed altered early-life growth, characterized by a faster tempo but lower specific early growth contribution according to the QEPS model. They also entered puberty earlier and experienced reduced puberty-specific height gain compared with the reference population. In contrast, basic ongoing growth was largely preserved, although shifted in time due to the earlier onset of puberty. Although basic growth during puberty was greater for boys with CAH-SW and girls with CAH-SV than in the reference population, this accounted for only a small difference in absolute height. Boys and girls with CAH, both SW and SV forms, were shorter at adult height (−1.7 SDS) relative to children in the reference population and to what was expected based on the stature of their own parents.

### 4.2 Benefit of using a growth model

A major strength of our study compared with other studies on growth patterns in patients with CAH was the use of a growth model as a mathematical tool. The QEPS model allowed us to analyze in detail and describe the growth pattern and interpret the functions of different growth periods of a child. The separated growth functions of QEPS, one for the specific early growth and another for the specific pubertal growth, make both descriptions more detailed. In addition, the model enables to study the growth influencing factors during different growth components, namely, hormonal substitution treatments. Childhood growth is known to be GH driven with permissive effect of cortisol and thyroid hormones, and pubertal growth–sex-steroid driven, with higher GH levels (21). However, QEPS is a mathematical tool to be used for interpretations of physiological growth patterns from early life to pubertal periods separated from the ongoing basic growth.

### 4.3 Phase-specific growth patterns in CAH

#### 4.3.1 Early-life growth (fetal and infancy period)

Previous research has demonstrated that prenatal hyperandrogenism affects size at birth in newborns with CAH, findings confirmed by our study with longer birth length in boys and girls with the SW form (22–25). Studies from Finland and Italy have reported that children with CAH were longer at birth than healthy children of the same ethnicity (24, 25). Data from the United Kingdom and Sweden, however, did not show differences between birth weight_SDS_ in girls and boys with CAH compared with national references (26, 27). In a German study on 116 CAH newborns, birth length and weight did not differ from the general population, were similar across the different CAH genotypes, and were not influenced by maternal age, mode of delivery, and maternal parity (23). In the present study, we found that newborns with CAH-SW of both sexes were longer but not heavier at birth than the healthy birth reference population of the healthiest 800,000 newborns out of 1 million born in Sweden in 1900–1999 (13). Compared postnatally with another healthy reference population, the GrowUp_1994_Gothenburg cohort born in 1990, boys with CAH were taller with a faster tempo of growth during early life from fetal life throughout the infancy period (18).

In line with our findings, a large longitudinal multicenter European study reported impaired growth of individuals with the SW form of CAH in infancy and early childhood (0–3 years of age), followed by normal growth patterns in childhood until puberty (10).

In contrast, children with CAH-SV had normal patterns of growth in infancy and early childhood and were considerably taller than healthy subjects during later childhood (10). Analysis of early growth and bone maturation of patients with delayed diagnosis of CAH-SV indicates relative androgen insensitivity during the first 6–12 months of life, predisposing to normal growth velocity during this time period (28, 29). After the age of 1 year, untreated CAH-SV patients will have a significant acceleration of bone maturation with increased growth rate. This observation has implications for the recommendation to identify individuals with classical CAH during the newborn screening program. It has been proposed to start low-dose hydrocortisone treatment in these CAH-SV patients during the first 6–12 months of life, with doses being adjusted thereafter to avoid accelerated skeletal maturation or growth deceleration (8).

Fetal growth is determined by genetic, maternal, and placental factors, and in the second part of pregnancy, the intrauterine environment, nutrition, and hormonal milieu are the most important factors (30).

Although the reason for the difference between the sexes in size at birth is not fully understood, studies suggest that the presence of the Y-chromosome and androgenic action in the prenatal period may play an important role (31). A previous study has shown that the degree of androgenization is directly related to birth weight, and this factor proved to be superior even to chromosomal sex (32).

#### 4.3.2 Childhood growth

A previous French study reported advanced bone age at 8 years of age in children with CAH as a strong risk factor for short adult height (33).

Early adrenarche is a significant factor contributing to growth alterations in patients with CAH. The excessive production of adrenal androgens during this period accelerates skeletal maturation, leading to advanced bone age. Early closure of epiphyseal growth plates results in a reduced potential for further longitudinal growth, thereby contributing to shorter adult height outcomes (31). Studies have demonstrated that untreated or poorly controlled androgen excess exacerbates this process, underscoring the need for optimal glucocorticoid therapy to mitigate these effects (34).

In contrast, a recent study found that patients with and without significant bone age advancement, and thus differing height prediction during adrenarche, showed similar (predicted) adult height when reassessed during their pubertal years (35).

Overall, early adrenarche contributes significantly to the growth challenges observed in CAH by advancing skeletal development prematurely and diminishing the overall height potential. Early and effective management of adrenal androgen levels is crucial to minimizing these long-term growth-related consequences (2, 36).

#### 4.3.3 Pubertal growth and timing

Both boys and girls with CAH had an earlier onset of puberty with less specific pubertal height gain than children in the reference population, despite showing more basic growth during puberty, resulting in shorter adult height, both when compared with a reference population and to their own parents. Height just before puberty, at onset of puberty, and gain during the pubertal years is important for adult height, with earlier onset/timing and sometimes less pubertal growth observed in patients with CAH compared with the general population (2, 36).

High androgen levels during prepubescence have been suggested to contribute to short adult height (2), which is related to hydrocortisone bioavailability in patients with 21-OH deficiency (37, 38). The multicenter Central European study identified peak height velocity (PHV) in both boys and girls with CAH as normal, but it occurred at an earlier age than in the general population (10).

The French study with large numbers of patients with classical CAH studied growth aspects during the pubertal years. They found pubertal growth to be almost absent in individuals with CAH and noted that mean adult height was shorter than that of the general population for both girls (−1.2 SDS, 156.7cm) and boys (−1.0 SDS, 168.8cm) (33). On the contrary, the before-mentioned multicenter Central European study showed children with SV and SW forms of CAH to have normal pubertal growth and a reduced adult height; however, adult height was reduced to a lesser extent than found in other studies (2, 8, 10, 39–41). Our results using QEPS functions showed less specific pubertal growth and more basic QES-growth resulting in a similar total amount of pubertal growth. Only girls with CAH-SV experienced a shorter duration of pubertal growth.

Early pubertal onset observed in patients with classical CAH is likely a key contributor to reduced adult height. Chronic exposure to excess adrenal androgens during childhood accelerates bone maturation through increased peripheral aromatization to estrogens, leading to earlier epiphyseal fusion and shortening of the growth period. In parallel, glucocorticoid treatment—while essential for disease control—may suppress the growth hormone–insulin-like growth factor 1 (GH–IGF-1) axis when administered in supraphysiological doses, further limiting linear growth potential (42).

The combined effects of androgen excess and glucocorticoid exposure may therefore result in a pattern characterized by earlier pubertal timing, reduced pubertal height gain, and compromised adult height (43). This mechanism differs in emphasis but not in principle from growth impairment observed in other endocrine disorders associated with early puberty or glucocorticoid excess, such as central precocious puberty or Cushing syndrome, where premature skeletal maturation and early epiphyseal closure play a central role (44).

The relationship between glucocorticoid dosage and pubertal development in CAH is complex. Higher glucocorticoid doses may reduce androgen excess and potentially delay pubertal onset; however, prolonged exposure to supraphysiological glucocorticoid levels is also associated with direct growth suppression and attenuation of pubertal height gain. Thus, impaired adult height may result not only from advanced skeletal maturation due to androgen excess but also from overtreatment-related growth inhibition, emphasizing the narrow therapeutic window in CAH management (45, 46).

Adjunctive treatment strategies, including growth hormone therapy, gonadotropin-releasing hormone analogs, or aromatase inhibitors, have been proposed in selected cases to preserve pubertal growth and optimize adult height. While early administration of growth hormone in combination with carefully calibrated glucocorticoid therapy may theoretically support linear growth, long-term safety, efficacy, and cost-effectiveness remain incompletely established. These interventions should therefore be considered on an individual basis and within specialized centers, particularly given the potential metabolic and skeletal risks associated with prolonged hormonal manipulation (42).

### 4.4 Adult height

The detailed analysis performed in our study showed that patients with CAH, both SW and SV forms, did not reach their MPH and were significantly shorter than both the reference population (by −1.7 SDS in boys and −1.6 SDS in girls) and their parents. In previous studies, patients with CAH were on average 12.1cm shorter than controls (36), or their adult height was below −2 SDS (47). We could also clearly show that the loss came mainly from the two specific growth functions, E for early life growth, and P for specific pubertal growth; the first is due to the disease, and the second might be due to treatment. Comparing HC doses between patient groups, it has become evident that to effectively manage the disease, some patients require a higher dose of hydrocortisone that goes beyond standard treatment recommendations. This finding emphasizes the need for personalized care and individualized dosages to achieve optimal health outcomes through long-term follow-up. By providing tailored treatment options, patients might benefit more appropriate care for better health outcomes. Additional treatment with gonadotropin analogs, aromatase inhibitors, or growth hormone may be beneficial in some selected cases (48, 49).

### 4.5 Strength and limitations

The main limitations of the current study were the retrospective study design and the relatively small number of included patients with CAH limiting validity of p-values <0.05 and making comparisons between subgroups difficult to interpret. The anthropometric measurements were performed by trained medical personnel in certified healthcare settings, including the tertiary referral center for patients diagnosed in infancy. Although the single-center design may limit generalizability, it also ensures consistency in treatment protocols and follow-up, thereby reducing variability and potential bias.

Comparing the Lithuanian CAH patient group with a Swedish reference population may also be considered as a weakness of the study design; however, this population was chosen in the absence of a formal growth reference for Lithuanian children. In Lithuania, it has been reported that the average full-term (39–40 gestation weeks) birth weights are 3.526–3.668 kg for men and 3.376–3.509 kg for women, with average birth lengths of 52.4–53.0 cm for men and 51.7–52.2 cm for women (19). These are broadly similar to the values in the present Swedish birth reference of 800,000 healthiest newborns born in 1990–1999 (14). Furthermore, the mean parental heights of the two different populations were remarkably similar, with mean differences of 5mm or less, indicating that comparisons are reasonable.

Also, one of the limitations is that the height measurements of the GrowUp_1974_ Gothenburg cohort during infancy and early childhood may be less precise due to their non-clinic settings.

A strength compared with other studies was the availability of data on the full pattern of growth from birth to adult height. A skilled study staff measured the reference population until adult height, with growth velocity below 0.5cm per year, and the CAH patients were measured by skilled staff of the pediatric endocrine department. Data on adult height, in particular, have been lacking in many earlier studies which have only reported on “near final” or “near adult” height, making their analyses and conclusions less precise.

Given the mentioned limitations, the study is positioned as exploratory and hypothesis-generating, focusing on accurate characterization of growth trajectories in classical CAH patients.

### 4.6 Future aspects

In future, it is important to confirm and extend the findings of the present study by applying the QEPS model to data from a larger number of patients with different forms of CAH. This will help to explore the impact of treatment regimen (and treatment options as modified release hydrocortisone or CRF receptor blocker) on growth patterns during the different growth periods. In turn, this will contribute to the development of prediction models for individualized treatment of CAH patients, similarly to successful prediction models for GH treatment (50).

## 5 Conclusion

For the first time, a detailed analysis of growth patterns from birth to adult height in individuals with CAH was made possible using the QEPS growth model. We found that patients with CAH had growth patterns that were distinct from those of a healthy reference population. Characteristic growth features in children with CAH included a greater birth length and a faster tempo of altered early-life growth during infancy. Patients with CAH also showed earlier pubertal onset and reduced specific pubertal growth, contributing to shorter adult height. This was obtained in all children with CAH independent of type, despite equal basic growth before puberty whereas reduced during puberty when compared with the reference population. The new knowledge obtained in this study holds the first step to be implemented in computerized monitoring tools for individualized GC treatment aiming for normalized growth through all growth periods, allowing patients to reach an adult height according to their genetical potential, as done for GH-substitution therapy (51, 52).

## Data Availability

The original contributions presented in the study are included in the article/supplementary material. Further inquiries can be directed to the corresponding authors.

## Glossary

17-OHP: 17-hydroxyprogesterone
AHSDS: standard deviation score of adult height, measured by the team, 0.5 cm/previous year.
BL: birth length
BMI: body mass index
BW: birth weight
CAH: congenital adrenal hyperplasia
CAH-SV: simple virilizing CAH form
CAH-SW: salt wasting CAH form
CI: confidence interval
DiffMPH BLSDS: difference between birth length and mid-parental height
Fhei: father height
GA: gestation age
GC: glucocorticoids
HC: hydrocortisone
MBR: Medical Birth Register
Mhei: mother height
MPH: mid-parental height
OR: odds ratio
PHV: peak height velocity
SD: standard deviation
SDS: standard deviation score
TargetH: target height
AgeP5: age at which 5% of the P-function growth is reached
AgeP50: age at which 50% of the P-function growth is reached
AgeP95: age at which 95% of the P-function growth is reached
AgeP100: age at which 100% of the P-function growth is reached
AgePPHV: age at peak height velocity of the P-function
AgeTEND: age at the end of puberty where the height velocity has decreased to 1 cm/y for function T' (age)
AgeTONSET: age at minimum height velocity of the T-function at start of the pubertal growth
AgeTPHV: age at peak height velocity of the T-function
Deltapubgain: Ppubgain minus QESpubgain
E: specific early-life growth function of age E(age) in cm
Emax: gain in adult height in cm due to E-function growth
Etsc: individual time scale ratio; modifying the time scale of the E-function growth
P: quadratic logistic function describing specific pubertal growth P(age) in cm
Pmax: pubertal gain in adult height in cm due to the P-function growth
Ppubgain: gain in height in cm due to the specific pubertal growth of the P-function from AgeP5 to AgeP100 = Pmax, P(AgeP5)=0.95*Pmax
Q: quadratic growth function of age Q(age) in cm
QE: sum of Q & E‐function giving non‐pubertal growth QE(age) in cm equal to total prepubertal height in cm as a function of age
Qmax: gain in adult height in cm due to Q-function growth
QESpubgain: gain in height in cm due to the pubertal growth of the QES-function from AgeP5 to AgeP100 = QESmax, QES(AgeP5)
S: stop function S(age) in cm, stopping the Q-function at the end of growth
Smax: reduction in adult height in cm due to stopping the Q-function at the end of growth
T: total height function in cm T(age) = QEPS(age) = Q(age) + E(age) + P(age) S(age)
Tmax: modeled total adult height in cm, Tmax = Qmax + Emax + Pmax - Smax
TmaxSDS: standard deviation score for Tmax
Tpubgain: total gain in height in cm from AgeP5 to AgeP100 = Ppubgain + QESpubgain
